# Risk Factors of Smartphone Addiction: A Systematic Review of Longitudinal Studies

**DOI:** 10.1002/puh2.202

**Published:** 2024-06-22

**Authors:** Sina Crowhurst, Hassan Hosseinzadeh

**Affiliations:** ^1^ School of Health & Society University of Wollongong Wollongong New South Wales Australia

**Keywords:** addiction, longitudinal studies, risk factors, smartphone, systematic review

## Abstract

**Background:**

Smartphone addiction is exponentially increasing worldwide. It has negative health consequences. Previous systematic reviews identified several risk factors of smartphone addiction; however, they were based on cross‐sectional data. This systematic review aimed to fill the gap by assessing smartphone addiction risk factors using longitudinal studies.

**Methods:**

This systematic review is registered with PROSPERO (CRD42023431529) and followed the Preferred Reporting Items for Systematic Reviews and Meta‐Analyses guidelines. Six databases, including Scopus, Medline, Web of Science, PubMed, ProQuest Central and PsycINFO, were searched to identify eligible studies. Studies were eligible if they assessed smartphone addiction as the outcome variable, were longitudinal and were published in English. All papers included in this review were assessed for the risk of bias and quality.

**Results:**

A total of 22 papers met the inclusion and exclusion criteria after the screening process. The results were categorised into three groups, including personal, social and environmental factors. Within the groups, seven risk factors, including mental health, emotions, academic stress, social rejection and peer victimisation as well as family dysfunction and parental phubbing, were identified. All of the risk factors were significant predictors of smartphone addiction. Mental health problems, social rejection and peer victimisation also displayed a bidirectional relationship with smartphone addiction. Inconsistent smartphone addiction measurements were used.

**Conclusion:**

This review has significant implications for policymakers as it identified seven risk factors for smartphone addiction. Further studies are warranted to improve the understanding of the aetiology of smartphone addiction and inform education, counselling and coping with smartphone addiction.

## Graphical abstract

This systematic review suggests that mental health, emotions, academic stress, social rejection and peer victimisation, as well as family dysfunction and parental phubbing, are main predictors of smartphone addiction. Mental health problems, social rejection and peer victimisation displayed a bidirectional relationship with smartphone addiction.

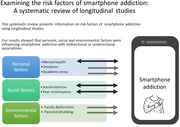

## 1 Background

The popularity of the smartphones is exponentially increasing worldwide. By the end of 2022, 68% of the world's population were smartphone users [1]. Smartphones have made our daily lives more convenient as they allow us to work online, engage with family and friends, take photos, search information and so much more [2]. Smartphones are the main platform for social networking, entertainment and web surfing nowadays [3]. Despite the benefits and convenience, concerns about the prevalence of problematic smartphone use are growing globally [4]. For instance, a recent study revealed that over 60% of the participants were addicted to their smartphone [5]. Smartphone use becomes problematic when it starts to interfere with daily life. Problematic smartphone use is often characterised by excessive smartphone use [6] or uncontrollable behaviours such as constantly checking for notifications [7]. Excessive or problematic smartphone use is viewed as a form of behavioural addiction, such as gaming addiction, internet addiction and overeating [7]. Smartphone addiction is associated with symptoms, such as withdrawal, mood dysregulation, cravings and a loss of control [8]. However, the term smartphone addiction is considered controversial, as some state that its addictive consequences do not meet the severity levels of those caused by drug addiction [9]. Nonetheless, problematic smartphone use and smartphone addiction are often used interchangeably [6]. For the purpose of consistency, the term smartphone addiction is used in this review.

Smartphone addiction is associated with negative health consequences, such as poor sleep quality, musculoskeletal problems and accidents [5, 10, 11] as well as pain and migraines [11]. Smartphone‐addicted individuals tend to have a more sedentary lifestyle [3, 10, 11], which has wide‐ranging adverse health consequences [12]. Furthermore, depression, anxiety, social anxiety [5, 10, 13], low self‐esteem and shyness [11] are the common psychological consequences of smartphone addiction. This evidence suggests that understanding the aetiology and predicting factors of smartphone addiction are essential to mitigate the health consequences and develop evidence‐based prevention strategies.

Previously, younger age, being female, hours spent on a smartphone as well as psychological factors, such as depressive symptoms, personality disorders and emotional coping strategies, have been associated with the development of smartphone addiction [14]. Smartphone ownership and high screen time patterns are not necessarily leading to smartphone addiction [4], but how and why the smartphone is used can lead to the addiction.

Previous systematic reviews synthesised aetiology evidence for smartphone addiction and categorised them into personal, interpersonal and behavioural factors [15]. However, they were based on cross‐sectional studies. This systematic review aims to fill this gap by examining the risk factors of smartphone addiction using longitudinal studies.

The importance of this research is twofold; first, smartphone addiction is continuously increasing globally [1]. Second, to date, there is no empirical evidence on the factors that influence or predict smartphone addiction using data from longitudinal studies [6, 15, 16].

## 2 Methods

### 2.1 Search Strategy

Six well‐known databases, including Scopus, Medline, Web of Science, PubMed, ProQuest Central and PsycINFO were searched to identify eligible studies. A variety of search terms, including ‘phone addict*’ OR ‘smartphone addict*’ OR ‘excessive smartphone use’ OR ‘excessive phone use’ OR ‘phone dependence’ OR ‘problematic phone use’ OR nomophobia, were used to generate a comprehensive result. As the smartphone gained popularity in 2008 [1], the literature was searched from January 2013 until February 2023. We believe that a minimum of 5 years would be enough to publish a longitudinal study. A detailed search strategy is provided in Table S1. All eligible studies were exported and managed in EndNote.

### 2.2 Inclusion and Exclusion Criteria

Only original longitudinal studies that explored smartphone addiction as the main outcome variable and were published in the English language were eligible for this review. There were no restrictions in terms of socio‐demographics or geographical areas. Studies were excluded if they had a study period of less than 6 months duration, were poorly written or did not offer a clear definition of risk factors.

### 2.3 Data Synthesis and Analysis

Data extraction included country study conducted, sample size, population, study duration, exposure and outcome measures (Table 1). The data extraction process was conducted manually using EndNote and Microsoft Excel (SC). The papers were reviewed and analysed independently by the authors based on the study inclusion and exclusion criteria (SC, HH). Disagreements were resolved by discussion. Data extraction and tabulation were completed by SC and then reviewed and checked by HH.

**TABLE 1:** Summary of study characteristics and main risk factors of smartphone addiction in the evaluation of longitudinal studies, 2019–2023.

Reference and country	Sample size	Type of population	Mean age/Age range	Gender	Covariates	Duration	Smartphone addiction assessment tool	Main risk factor
[37] China	1820	Adolescents	Mage 12.32 years	55.89% male 44.11% female	Gender, age, family SES, PSU scores, school, engagement	3 waves 1 year	Mobile phone addiction index	Childhood emotional neglect
[33] China	1820	Adolescents	Mage 12.32 years	55.89% male 44.11% female	Gender, age, family SES	3 waves 1 year	Mobile phone addiction index	Peer victimisation
[19] China	1181	Adolescents to young adults	Mage 18.91	49.3% males 50.7% females	Age Gender	2 waves 1 year	Mobile phone addiction tendency scale	Depressive symptoms
[38] China	890	Adolescents	Mage 15.9 years	49.0% males 51.0% females	Gender Place of residence T1 PSU	2 waves 6 months	Smartphone application‐based addiction scale	Childhood maltreatment
[34] China	1447	Adolescents	Mage 16.15 years	39.5% males 60.5% females	Gender	2 waves 6 months	Smartphone addiction scale	Parental phubbing
[35] China	1721	Adolescents	Mage 13.39 years	48.6% males 51.4% females	Age Gender	2 waves 6 months	Mobile phone problem use scale	Parental phubbing
[30] China	633	Adolescents	Mage 13.6 years	43.6% males 56.5% females	Age Gender	3 waves 1.5 years	Mobile phone problem use scale	Academic procrastination
[27] China	358	Adolescents	Mage 13.19 years	43.0% males 57.0% females	None	3 waves 2 years	Mobile phone problem use scale – short version	Autonomy needs dissatisfaction
[28] China	906	Adolescents	Mage 11.2 years	49% males 51% females	None	2 waves 1 year	Brief smartphone addiction scale	Loneliness
[20] China	902	Adolescents Young adults	19–21 years	N/M	Age Gender SES	2 waves 1 year	Mobile phone involvement questionnaire	Depression Anxiety
[39] China	2548	Adolescents and their parents	10–16 years	51.7% males 48.3% females	Parental education, family income, gender	3 waves 2 years	Smartphone addiction proneness scale	Poor parent child relationship
[32] China	1368	Adolescents	Mage 15 years	60.1% males 39.9% females	Age Family SES	3 waves 6 months	Mobile phone addiction index	Social rejection
[21] China	3827	Adolescents Young adults	Mage 18.87 years	52.8% males 47.2% females	Age Gender SES	4 waves 2 years	Smartphone addiction scale – short version	Depression
[40] China	2128	Children and adolescents	Mage 10.91 years	55.69% males 44.31% females	Gender, grade, T1 PSU	2 waves 10 months	Mobile phone problem use scale	Parental psychological control
[36] China	2260	Adolescents	Mage 12.76 years	49.65% males 50.35% females	Parental education, family income, age	2 waves 1 year	Smartphone addiction scale – short version	Parental phubbing
[22] China	124	Adolescents	Not mentioned	49% males 51% females	Gender	3 waves 2 years	Mobile phone addiction index	Stressful life events
[31] China	642	Adolescents	11–17 years	41.6% males 57.6% females 0.8% no gender	Gender, grade	4 waves 1.5 years	Mobile phone problem use scale	Academic stress
[23] China	34 1	Adolescents Young adults	Mage 21.24	24.3% males 75.7% females	Gender, age	3 waves 1 year	Smartphone addiction scale – short version	Depression
[24] China	1186	Adolescents	Not stated	47.7% males 52.3% females	Gender, age, parents’ education level	2 waves 1 year	Smartphone addiction scale – short version	Depression
[29] China	352	Adolescents Young adults	Mage 19.30 years	44.9% males 55.1% females	Gender	2 waves 8 months	Mobile phone addiction tendency scale	Boredom proneness
[25] China	197	Young adults	Not mentioned	41.1% males 58.9% females	Gender, age, major	3 waves 1 year	Mobile phone addiction index	Stressful life events
[26] China	313	Adolescents	14–18 years	36.1% males 63.9% females	Age, gender	2 waves 6 months	Smartphone addiction inventory	Depression

### 2.4 Reporting

**FIGURE 1:**
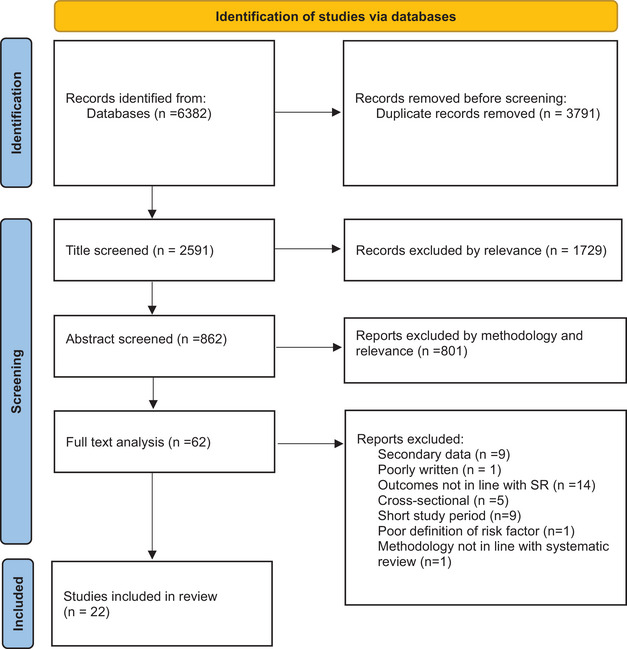
Preferred Reporting Items for Systematic Reviews and Meta‐Analyses (PRISMA) flow diagram for the systematic review of risk factors for smartphone addiction.

The protocol of this systematic review is registered in PROSPERO (CRD42023431529) and followed the PRISMA (Preferred Reporting Items for Systematic Reviews and Meta‐Analyses) guidelines [17] (Figure 1). The quality of the eligible papers was assessed by using the NIH (Quality Assessment of Systematic Reviews and Meta‐Analyses) [18].

### 2.5 Ethical Considerations

This is a systematic review study and does not require ethics approval. However, all the included studies stated that they were approved by a research ethics committee. All the included studies had an adequate sample size with appropriate outcome variables. Further, the quality of the eligible papers was assessed by using the NIH (Quality Assessment of Systematic Reviews and Meta‐Analyses) [18].

## 3 Results

A total of 6382 potential studies were identified through the database search (Figure 1). A total of 3791 duplicated papers were removed using the EndNote X9 functionality as well as manually. After title screening, 1729 papers were removed. The abstracts of the remaining papers (862) were screened for further scrutiny, and 61 papers remained for full text review (Table S3). Finally, 22 papers met the inclusion criteria for this review (Figure 1).

### 3.1 Risk of Bias Assessment

The quality of the 22 selected studies was assessed using the ‘Quality Assessment Tool for Observational Cohort and Cross‐sectional Studies’ developed by the National Institute of Health (NIH) [18]. This assessment tool is designed to assess the internal validity of the studies, testing for potential flaws in the study design, methodology or implementation and selection bias, including information bias, measurement bias and confounding factors.

The tool rates studies as ‘good’, ‘fair’ or ‘poor’. Studies are rated ‘good’ if more than 75% of the criteria in the assessment tool are addressed adequately; studies addressing 50%–75% of the criteria receive a ‘fair’ rating, and studies unable to satisfactorily answer 50% of the criteria receive a rating of ‘poor’.

From the 22 papers selected in this review, 5 [5] studies were considered good, 14 [13] were fair and 4 [4] were poor (Table 2); the full assessments are presented in Table S2. Our findings indicated that 23% of the selected papers had a low risk of bias, 59% had a moderate risk of bias and 18% had a high risk of bias. The poor ratings were mostly due to inadequate descriptions of study population, high attrition rate and invalidated outcome measurement tools. Further information can be provided upon request.

**TABLE 2:** Summary of study details, main findings and quality of the studies included in the evaluation of smartphone addiction with longitudinal studies, 2019–2023.

	Risk factors	Reference and location	Study details	Main findings	Quality
**Personal**	Mental health	[22] China	This study explored the relationship between stressful life events and smartphone addiction. A total of 124 adolescents participated in a 3‐wave study over a period of 2 years	Stressful life events predict subsequent smartphone addiction Depressive symptoms mediate the relationship between stressful life events and smartphone addiction	Poor
		[25] China	This study investigated the relationship between stressful life events and smartphone addiction, with the mediating roles of mental health problems. A total of 197 university students participated in a 3‐wave study over a 1‐year period	Stressful life events significantly predicted smartphone addiction Depressive symptoms, sleep quality and suicidal ideation fully mediated the association between stressful life events and smartphone addiction	Good
		[23] China	This study examined the relationship between depression and smartphone addiction. A total of 341 university students participated in a 3‐wave study over a 1‐year period	Depressive symptoms predicted smartphone addiction	Fair
		[20] China	This study evaluated the relationship between smartphone addiction and mental distress. A total of 902 participants participated in a 2‐wave study over a 1‐year period	Depression and anxiety predicted smartphone addiction Smartphone addiction did not predict depression and anxiety	Fair
		[26] China	This study tested the relationships between depression and smartphone addiction. A total of 313 high adolescents participated in a 2‐wave study over a 6‐month period	Depression predicted smartphone addiction Smartphone addiction did not predict depression	Poor
		[19] China	This study evaluated the longitudinal relationship between depression and smartphone addiction. A total of 1181 adolescents completed 2 waves of questionnaires over a 1‐year period	Depressive symptoms predicted smartphone addiction Smartphone addiction predicted depressive symptoms Bidirectional relationship	Fair
		[21] China	This study tested the relationship among smartphone addiction, loneliness and depressive symptoms. A total of 3827 college students participated in a 4‐wave study over a 2‐year period	Bidirectional relationship between smartphone addiction and depressive symptoms Loneliness mediated the association between smartphone addiction and depressive symptoms Gender differences were not found in these relationships	Good
		[24] China	This study examined the bidirectional relationship between smartphone addiction and depression. A total of 1186 adolescents participated in a 2‐wave study over a 1‐year period	Female group showed a significant bidirectional association between smartphone addiction and depression Male group showed no predictive effect between smartphone addiction and depression	Fair
	Emotions	[27]	This study examined the relationship between autonomy needs dissatisfaction and smartphone addiction, with boredom proneness mediators. A total of 358 adolescents participated in a 3‐wave study over a 2‐year period	Autonomy needs dissatisfaction predicted smartphone addiction Boredom proneness mediated the relationship between autonomy need dissatisfaction and smartphone addiction	Fair
		[28] China	This study evaluated the bidirectional relationship between loneliness and smartphone addiction. A total of 906 adolescents participated in a 2‐wave study over a 1‐year period	Trait loneliness positively predicted smartphone addiction Smartphone addiction did not predict loneliness	Fair
		[29] China	This study examined the bidirectional relationship between boredom proneness and smartphone addiction. A total of 352 adolescents and young adults participated in a 2‐wave study over an 8‐month period	Boredom proneness significantly predicted smartphone addiction Smartphone addiction significantly predicted boredom proneness	Good
	Academic stress	[30] China	This study investigated the reciprocal relationship between academic procrastination and smartphone addiction. A total of 633 adolescents participated in a 3‐wave longitudinal study over the course of 1.5 years	Academic procrastination positively predicted subsequent smartphone addiction Smartphone addiction did not stably predict academic procrastination	Fair
		[31] China	This study examined the association between academic stress and smartphone addiction. A total of 642 adolescents completed the 3‐wave longitudinal study over a 1.5‐year period	Academic stress positively predicted smartphone addiction	Fair
**Social**	Social rejection	[32] China	This study examined the reciprocal relationship between social redaction and smartphone addiction. A total of 1368 adolescents participated in the 3‐wave longitudinal study over a 6‐month period	Social rejection predicts smartphone addiction Smartphone addiction predicts social rejection Reciprocal relationship	Poor
	Peer victimisation	[33]	This study examined the reciprocal relationship between peer victimisation and smartphone addiction. A total of 1820 adolescents participated in a 3‐wave study over the period of 1 year	Peer victimisation positively predicted smartphone addiction Smartphone addiction positively predicted peer victimisation Reciprocal relationship	Fair
	Childhood experiences	[37] China	This study examined the relationship between childhood emotional neglect and smartphone addiction. A total of 1987 adolescents participated in a 3‐wave study over the period of 1 year	Childhood emotional neglect positively predicted smartphone addiction	Good
		[38] China	This study examined the relationship between childhood maltreatment and smartphone addiction. A total of 890 participants completed a 2‐wave study over the period of 6 months	Childhood maltreatment positively predicted smartphone addiction	Good
		[39] China	This study examined relationship between parent–child relationship and subsequent smartphone addiction. A total of 2548 adolescents and their parents participated in a 3‐wave study over a time period of 2 years	Poor parent–child relationship predicted smartphone addiction	Fair
		[40] China	This study examined the relationship between parental psychological control and smartphone addiction. A total of 2128 participants contributed to a 2‐wave study over the period of 8 months	Parental psychological control predicts subsequent smartphone addiction Smartphone addiction predicts parental psychological control Reciprocal relationship	Fair
	Parental phubbing	[34] China	This study examined the relationship between parental phubbing and subsequent smartphone addiction. A total of 1447 adolescents participated in the 2‐wave longitudinal study	Parental phubbing predicted adolescents’ subsequent smartphone addiction	Fair
		[36] China	This study examined the reciprocal relationship between parental pubbing and smartphone addiction. A total of 2260 adolescents participated in the 2‐wave study, with a time interval of 1 year	Parental phubbing predicted smartphone addiction; the reverse was not true Smartphone addiction did not predict parental phubbing	Fair
		[35]	This study examined the relationship between parental phubbing and smartphone addiction. A total of 1721 adolescents participated in the 2‐wave study over a time frame of 6 months	Parental phubbing predicted smartphone addiction	Poor

### 3.2 Study Characteristics

The selected studies reported that personal, social and environmental factors were contributing to smartphone addiction. Personal factors included mental health [19, 20, 21, 22, 23, 24, 25, 26], emotions [27, 28, 29] and academic stress [30, 31]. Social factors included social rejection [32] and peer victimisation [33]. Environmental factors included parental phubbing [34, 35, 36] and family dysfunction [37, 38, 39, 40] (Table 2). Sample sizes ranged from 124 [22] to 3827 [21] participants. Although no age restrictions were applied during the search, participants’ ages ranged from 10 to 23 years old, and the majority were adolescents (Table 1). Despite the fact that there were no geographical restrictions on the search, all 22 studies were conducted in China (Tables 1 and 2).

Smartphone addiction was measured using nine different scales (Table 1). The reliability levels of the scales were good, with Cronbach's alpha score of 0.8 or higher [41]. Four out of the 10 assessment tools used, including mobile phone addiction index [42], smartphone addiction scale [43], smartphone addiction scale–short‐version [44], mobile phone problem use scale [45] and mobile phone addiction tendency scale [46], had adequate consistency and validity. The remaining six assessment tools’ validity was not assessed.

### 3.3 Personal Factors

#### 3.3.1 Mental Health

A total of eight studies assessed the relationship between mental health and smartphone addiction. Five studies found a predictive relationship between mental health problems and subsequent smartphone addiction. Two studies indicated that stressful life events predicted smartphone addiction; this relationship was mediated by depressive symptoms [22, 25], sleep quality and suicidal ideation [25]. Three studies indicated that depressive symptoms [20, 23, 26] and anxiety [20] significantly predict smartphone addiction. However, three studies found bidirectional relationships among depressive symptoms [19, 21], loneliness [21] and smartphone addiction. One study [24] found a bidirectional relationship between smartphone addiction and depression; however, multi‐group analysis revealed that this was only true for the female population, and the association was not significant for males.

#### 3.3.2 Emotions

A total of three studies assessed the relationship between emotions and smartphone addiction. One study [27] found that autonomy need dissatisfaction predicted smartphone addiction, and it was mediated by boredom proneness. Another study [28] revealed that trait loneliness positively predicted smartphone addiction. Lastly, boredom proneness and smartphone addiction displayed a reciprocal relationship [29].

#### 3.3.3 Academic Stress

Two studies found that academic procrastination [30] and academic stress [31] are positively associated with smartphone addiction.

### 3.4 Social Factors

#### 3.4.1 Social Rejection

Two studies assessed the relationship between social factors and smartphone addiction. One study [32] indicated a reciprocal relationship between social rejection and smartphone addiction, where social rejection significantly predicted smartphone addiction and vice versa. Another study [33] found a reciprocal relationship between peer victimisation and smartphone addiction.

### 3.5 Environmental

#### 3.5.1 Family Dysfunction

A total of four studies examined the relationship between family dysfunction and smartphone addiction. Two of them found that childhood emotional neglect [37] and childhood maltreatment [38] positively predicted smartphone addiction. One study showed that adolescents with a poor parent–child relationship had a higher tendency for smartphone addiction [39]. Lastly, one study found a reciprocal relationship between parental psychological control and smartphone addiction [40].

#### 3.5.2 Parental Phubbing

Three studies examined the relationship between parental phubbing and smartphone addiction. All three indicated that parental phubbing predicts smartphone addiction [34, 35, 36].

## 4 Discussion

Previously, only one systematic review [15] summarised the evidence on predictive risk factors for smartphone addiction. However, it was based on cross‐sectional data. This systematic review aimed to fill this gap by examining the risk factors for smartphone addiction using longitudinal studies. To the best of our knowledge, this is the first systematic review to utilise empirical evidence from longitudinal studies that provide evidence on the predictive risk factors of smartphone addiction. Out of 6382 potential studies, 22 met the inclusion criteria for this review. Our findings identified seven personal, social and environmental risk factors. However, some of the risk factors generated a bidirectional relationship with smartphone addiction. Thus, it is important to discuss the bidirectionality of those risk factors to better understand the aetiology of smartphone addiction.

### 4.1 Personal Factors

Our results showed that poor mental health indicates an overall predictive relationship with smartphone addiction [20, 22, 23, 25, 26]; this is consistent with previous research, showing that mental health problems such as depressive symptoms and anxiety are predictors of smartphone addiction [47, 48, 49]. Three studies found a bidirectional relationship between mental health and smartphone addiction [19, 21, 24]; this was also indicated by previous research reporting that depressive symptoms and smartphone addiction display a reciprocal relationship [50, 51]. This finding might be explained by the fact that individual suffering from mental health problems uses a smartphone as a coping strategy to escape negative emotions [22].

Further, individuals experiencing depressive symptoms are more likely to rely on a smartphone to alleviate their negative feelings [23, 52]. Specifically, a smartphone may provide individual suffering from a mental health problem with a convenient means for distraction, allowing them to escape negative emotions [20, 26]. However, previous studies have shown that smartphone addiction does not relieve depressive symptoms but rather worsens them [50], which suggests a bidirectional relationship between smartphone addiction and mental health problems. These results highlight an interconnection between mental health conditions and smartphone addiction. Thus, future research and reviews should focus on establishing a more comprehensive understanding of the aetiology and long‐term associations between mental health and smartphone addiction. Furthermore, it is critical to prioritise individuals suffering from mental health problems in any prevention interventions focusing on reducing smartphone addiction. Social support and family functioning [53], mindfulness training [54] and improving in‐person communication [52] might also help ease the use of a smartphone to cope with depressive symptoms.

In line with literature [55, 56, 57], our results revealed that emotions such as autonomy need dissatisfaction [27], trait loneliness [28] and boredom proneness [29] predicted smartphone addiction. This may be because adolescents who lack the need for autonomy and relatedness [58] may engage in ameliorating behaviours, such as engaging with the internet [59] through their smartphone, to compensate for the lack of fulfilment for their need for autonomy and social interactions and amusement [27, 28, 60], which inherently increases the risk for smartphone addiction. Excessive parental restrictions on children's online behaviours may cause further frustration in the need for autonomy and may increase addiction‐like tendencies [61]. Thus, supporting children's autonomy and collaboratively setting boundaries may mitigate the negative effects of excessive smartphone use [62].

Our results indicated that boredom proneness was both a predictor [27, 29] and a result of smartphone addiction [29]. This maybe because boredom‐prone individuals may use their smartphones to engage in interesting and challenging stimuli. Further, long‐term excessive use of smartphones may lead to overstimulation [63], making them insensitive to the stimuli and ultimately making people more and more prone to boredom. Our results showed that academic procrastination [30] and academic stress [31] were significant predictors of smartphone addiction. This is consistent with previous cross‐sectional studies for procrastination [64, 65, 66] and academic stress [66, 67], respectively.

This may be because academic procrastinators unnecessarily postpone learning tasks [68], which potentially provides opportunities to participate in distracting activities, such as engaging with their smartphone [30], for entertainment or to relieve negative emotions [31, 60, 69]. This can provide temporary relief from academic pressure [69] but has the potential to develop into habits to avoid academic tasks [30]. Moreover, previous literature found that internet addiction reinforces procrastination behaviours and severely reduces academic performance of adolescents [60], which may lead to increased academic stress [70], a vicious cycle of self‐reinforcement. Literature suggests that positive parent–child relationship [71], parental supervision and authoritative parenting behaviour [72], as well as friendship satisfaction and academic motivation [73], can be protective factors for the addictive use of smartphones. Moreover, available evidence suggests that establishing skills, such as planning, self‐monitoring, time management and proactive attitudes, as well as emotional awareness and emotional regulation in stressful situations, are effective strategies to improve and prevent procrastination behaviours [74]. Thus, prevention strategies should focus on incorporating these methods to enhance students’ academic performance.

### 4.2 Social Factors

Social factors displayed a reciprocal relationship with smartphone addiction [32, 33] and highlighted a continuous cycle through negative reinforcement. In line with our finding, previously, studies indicated a predictive relationship of social rejection [75, 76] and peer victimisation [77] with smartphone addiction. This finding suggests that adolescents with the unmet psychological need for relatedness [58] and the feeling of belonging [60] due to social rejection and peer victimisation [75] might turn to virtual relationships via smartphones [32]. This may replace face‐to‐face interactions but provides adolescents with the options to maintain virtual social interactions while avoiding negative social experiences, which may exacerbate smartphone addiction through negative reinforcement [78]. Although the excessive engaging with smartphone might bring short‐term fulfilment, the reciprocal relationship indicated that excessive smartphone use further aggravates the problem. In fact, engaging with social network service significantly increased the risk for smartphone addiction [79]. This highlights the importance of adequate social supports to adolescents [80].

### 4.3 Environmental Factors

Our results indicated that family dysfunction was a significant predictor of smartphone addiction [37, 38, 39, 40]. This is consistent with similar previous research, indicating that domestic violence and parental addiction [81], parental neglect [82], childhood maltreatment [83] and childhood trauma [84] are significantly associated with smartphone addiction. This finding suggests that adolescents may use their smartphones as a coping strategy [59] by connecting with friends and enjoying entertainment online to compensate for family dysfunction [40]. However, this requires further studies to determine how family dysfunction contributes to smartphone addiction.

Our finding showing a reciprocal relationship between parental psychological control and smartphone addiction suggests that parents may exert psychological control to limit the amount of time spent on the smartphone [40], but it is more likely to further frustrate psychological needs and decrease adolescents’ psychological security in a continuous cycle of negative reinforcement. Previous studies indicated that the lower the psychological security of adolescents, the more they depend on smartphones to regulate psychological needs and improve their psychological security [85].

Parental phubbing also significantly predicted smartphone addiction [34, 35, 36] without a reciprocal relationship [36], confirming findings of previous cross‐sectional studies [86, 87, 88]. According to the social learning theory [89], adolescents exposed to parents overusing their smartphones are inclined to copy their parents’ behaviour, as they may see it as a social norm. As such, our findings suggest that parental phubbing may set a bad example for children and adolescents’ observational learning and may increase their risk of smartphone addiction [87]. That is, adolescents receiving higher levels of parental phubbing are more likely to develop smartphone addiction.

Overall, childhood family environment was a significant factor in the development of smartphone addiction in adolescents [39]. This is in line with literature showing that children with positive parent–child relationships are less likely to engage in smartphone addiction behaviours [71, 90]. Thus, to decrease the risk of smartphone addiction, it is important to bring awareness to the concept of parental phubbing [39].

This review offers invaluable longitudinal evidence on personal, social and environmental factors that predict smartphone addiction, confirming findings of previous cross‐sectional studies. This adds significant value to the current body of literature. Our results were able to establish that mental health, emotions, academic stress, parental phubbing and family dysfunction are significant predictors of smartphone addiction. We found that social factors, mental health and smartphone addiction form bidirectional relationships, which emphasise the interconnection and negative reinforcements of all involved factors. With these results, we are able to make justified recommendations for future research.

### 4.4 Limitations

Despite offering invaluable information about smartphone addiction, this review has some limitations which deserve to be mentioned. First, despite no geographical restrictions during the literature search, all 22 studies included in this review were conducted in China. Due to cultural differences, results obtained from this review might not be applicable to Western counterparts, especially for risk factors such as academic stress, due to the extreme competition for future education prospects [69]. Furthermore, China is a collectivist country with a high emphasis on social relations and interpersonal relationships; thus, negative social interactions may have a greater impact on adolescents [32]. This emphasises the urgent need for further longitudinal studies across a variety of age groups, countries and cultures to establish a more comprehensive understanding of the aetiology of smartphone addiction.

Second, despite having no age restrictions during the literature search, the majority of participants were adolescents (Table 1). This may be because children and adolescents are at the highest risk of smartphone‐related addictive behaviour [91, 92], and the transition from adolescence to emerging adulthood has been shown to be an important period for the establishment of risky behaviour patterns [93].

Furthermore, all studies in this systematic review relied on self‐reported data, which is commonly used in this field of research. However, self‐reported data can be affected by an external bias caused by social desirability or approval bias [94]. As the perception of excessive smartphone use can vary considerably between individuals, the true value of mobile phone usage is either over‐ or understated.

Additionally, there are significant inconsistencies in the tool used to assess smartphone addiction; this can have implications in the overall reliability and validity of the results. Overall, 9 different assessment tools were used across 22 studies (Table 1). This was especially evident in the mental health section, where five different tools were used across nine studies. Thus, we make the following recommendations, first, conduct an analysis and review of the smartphone addiction assessment tools to determine comparability and/or overlap between the key themes of the assessment tools; second, use the information to develop a standardised and validated smartphone addiction assessment tool; and third, include objective measures such as monitoring apps to measure smartphone addiction accurately and consistently. Due to these inconsistencies, a meta‐analysis was not conducted to avoid biases.

## 5 Conclusion

This systematic review offers invaluable information about the personal, social and environmental factors impacting smartphone addiction by synthesising and analysing more recent longitudinal evidence. Our results confirm the findings of previous cross‐sectional studies and suggest that emotions, academic stress, family dysfunction and parental phubbing are the main predictors of smartphone addiction. Interestingly, social rejection and peer victimisation displayed a bidirectional relationship with smartphone addiction. This has significant implications for decision makers and suggests that smartphone addiction and its predicting factors might be interconnected and reinforce each other. Importantly, our findings showed that there are inconsistencies in the terms of the relationship between mental health and smartphone addiction. Some studies found predictive relationships, whereas others suggested bidirectional relationships. Furthermore, this review identified that various assessment tools were used for determining smartphone addiction. This suggests that inconsistencies in assessment tools may impact the consistency and validity of the factors predicting smartphone addiction.

## Author Contributions

**Sina Crowhurst**: writing–original draft. **Hassan Hosseinzadeh**: writing–review and editing; supervision.

## Conflicts of Interest

The authors declare no conflicts of interest.

## Supporting information

Supporting Information

Supporting Information

Supporting Information

## Data Availability

The data that support the findings of this systematic review are available on request from the corresponding author.
