# Supplementary material for: Risk Factors of Smartphone Addiction: A Systematic Review of Longitudinal Studies
Source: Public Health Chall. 2024 Jun 22;3(2):e202. doi: 10.1002/puh2.202 (PMC12039634; doi:10.1002/puh2.202)
Supplement: Supplementary file 1 — Supporting Information [file PUH2-3-e202-s001.docx]

**Supplementary Table 1 - Search history**

| Database | Type of search | Search terms | Limiters | Yield |
| --- | --- | --- | --- | --- |
| Medline: | Title and abstract search  (select a field (optional)) | "phone addict*" OR “smartphone addict*” OR “excessive smartphone use” OR "excessive phone use" OR “phone dependence" OR "problematic phone use" OR nomophobia | Limiters: Date of Publication: 2013-2023; English Language; Publication Type: Journal Article; Scholarly (Peer Reviewed) Journals | 916 |
| APA PsycInfo: | Title and abstract search  (select a field (optional)) | Same as above | Publication Year: 2013-2023; Peer Reviewed; Publication Type: Peer Reviewed Journal; English; Methodology: quantitative study | 579 |
| Scopus: | Title and abstract search  (TITLE-ABS-KEY) | Same as above | English language, articles, publication last 10 years | 1577 |
| Web of science | Title and abstract search  (Topic) | Same as above | Publication years: 2013-2023; English Language; document type article, exclude review articles | 1720 |
| Pubmed: | Title and abstract search  [All Fields] | Same as above | Publication years: 2013-2023; English Language | 941 |
| ProQuest central: | Title and abstract search  (Noft) | Same as above | Scholarly Journals, 2013 – 2023, Article, English | 649 |

Limiters were slightly different in each data base due to their functionality
