# Supplementary material for: Risk Factors of Smartphone Addiction: A Systematic Review of Longitudinal Studies
Source: Public Health Chall. 2024 Jun 22;3(2):e202. doi: 10.1002/puh2.202 (PMC12039634; doi:10.1002/puh2.202)
Supplement: Supplementary file 2 — Supporting Information [file PUH2-3-e202-s002.docx]

**Supplementary Table 2. Quality Assessments**

| Questions – see details below | | | | | | | | | | | | | | | |
| --- | --- | --- | --- | --- | --- | --- | --- | --- | --- | --- | --- | --- | --- | --- | --- |
| Reference | 1 | 2 | 3 | 4 | 5 | 6 | 7 | 8 | 9 | 10 | 11 | 12 | 13 | 14 | Rating |
| (Chen, Zhang, et al. 2021) | Yes | Yes | No | Yes | No | Yes | Yes | N/A | Yes | No | Yes | N/A | Yes | Yes | Good |
| (Chen, Zhu, et al. 2021) | Yes | Yes | No | Yes | No | Yes | Yes | N/A | No | Yes | Yes | N/A | Yes | No | Fair |
| (Cui et al. 2021) | Yes | Yes | No | Yes | No | Yes | Yes | N/A | No | Yes | Yes | N/A | Yes | No | Fair |
| (Geng et al. 2022) | Yes | Yes | No | Yes | Yes | Yes | No | N/A | Yes | Yes | No | N/A | Yes | Yes | Good |
| (Geng et al. 2021) | Yes | Yes | No | Yes | No | Yes | No | N/A | Yes | No | Yes | N/A | Yes | No | Fair |
| (Hong et al. 2019) | Yes | Yes | No | Yes | No | Yes | No | N/A | No | No | No | N/A | No | No | Poor*** |
| (Hong et al. 2021) | Yes | Yes | No | Yes | No | Yes | Yes | N/A | Yes | Yes | Yes | N/A | No | No | Fair |
| (Hong et al. 2020) | Yes | Yes | No | Yes | No | Yes | Yes | N/A | No | No | Yes | N/A | No | No | Fair |
| (Hu & Xiang 2022) | Yes | Yes | No | Yes | Yes | Yes | Yes | N/A | No | No | No | N/A | No | No | Fair |
| (Kang et al. 2020) | Yes | Yes | No | Yes | No | Yes | Yes | N/A | No | Yes | No | N/A | Yes | No | Fair |
| (Lai et al. 2022) | Yes | Yes | No | Yes | No | Yes | Yes | N/A | No | Yes | No | N/A | Yes | No | Fair |
| (Li et al. 2023) | Yes | No | No | No | No | Yes | No | N/A | Yes | Yes | Yes | N/A | No | No | Poor* |
| (Shi et al. 2023) | Yes | Yes | No | Yes | No | Yes | Yes | N/A | Yes | Yes | Yes | N/A | Yes | No | Good |
| (Wang et al. 2022) | Yes | Yes | No | Yes | No | Yes | Yes | N/A | No | No | Yes | N/A | Yes | Yes | Fair |
| (Wang et al. 2023) | Yes | Yes | No | Yes | No | Yes | Yes | N/A | No | No | Yes | N/A | Yes | Yes | Fair |
| (Xie et al. 2020) | Yes | Yes | No | Yes | No | Yes | Yes | N/A | No | No | No | N/A | No | No | Poor*** |
| (Yang et al. 2022) | Yes | Yes | No | Yes | No | Yes | Yes | N/A | Yes | No | Yes | N/A | Yes | No | Fair |
| (Yuan et al. 2021) | Yes | Yes | No | Yes | No | Yes | No | N/A | Yes | No | Yes | N/A | No* | No | Fair** |
| (Zhang et al. 2023) | Yes | Yes | No | Yes | No | Yes | Yes | N/A | No | No | Yes | N/A | Yes | No | Fair |
| (Zhang et al. 2022) | Yes | Yes | No | Yes | No | Yes | Yes | N/A | Yes | Yes | Yes | N/A | Yes | No | Good |
| (Zhao et al. 2021) | Yes | Yes | No | Yes | No | Yes | Yes | N/A | Yes | Yes | Yes | N/A | Yes | No | Good |
| (Zhou et al. 2021) | Yes | Yes | No | Yes | No | Yes | No | N/A | No | No | No | N/A | No | No | Poor*** |

*No description of the study population, no reference to attrition rate, **high attrition rate, 82% *** High attrition rate, exposure and outcome measures have not been validated

Clarifications for this assessment:

Question 9 and 11: All studies in this systematic review utilised self-report data as their assessment tool. Self-reported data can be affected by an external bias caused by social desirability or approval bias. However, for the purpose of these assessments, the questions are answered with yes if the data collection tool has been assessed for its validity and this is referenced in the article and has a Cronbach’s alphas score of 0.8 and above. Considering that the remaining criteria are also met.

Question 14: For the purpose of this assessment, confounders are classified as gender and SES (Durak 2019) and control for T1 PSU as discussed in the main text.

Rating: Studies that answered yes to less than six (50%) of the questions received a rating of poor, studies that answered yes to six to eight (50-75%) received a fair, studies that answered yes to nine (75%) and above received good.

Questions:

1. Was the research question or objective in this paper clearly stated?

2. Was the study population clearly specified and defined?

3. Was the participation rate of eligible persons at least 50%?

4. Were all the subjects selected or recruited from the same or similar populations (including the same time period)? Were inclusion and exclusion criteria for being in the study prespecified and applied uniformly to all participants?

5. Was a sample size justification, power description, or variance and effect estimates provided?

6. For the analyses in this paper, were the exposure(s) of interest measured prior to the outcome(s) being measured?

7. Was the timeframe sufficient so that one could reasonably expect to see an association between exposure and outcome if it existed?

8. For exposures that can vary in amount or level, did the study examine different levels of the exposure as related to the outcome (e.g., categories of exposure, or exposure measured as continuous variable)?

9. Were the exposure measures (independent variables) clearly defined, valid, reliable, and implemented consistently across all study participants?

10. Was the exposure(s) assessed more than once over time?

11. Were the outcome measures (dependent variables) clearly defined, valid, reliable, and implemented consistently across all study participants?

12. Were the outcome assessors blinded to the exposure status of participants?

13. Was loss to follow-up after baseline 20% or less?

14. Were key potential confounding variables measured and adjusted statistically for their impact on the relationship between exposure(s) and outcome(s)?

Chen, Y, Zhang, Y, Zhang, L, Luo, F, Xu, W, Huang, J, Yang, L & Zhang, W 2021, ‘Childhood emotional neglect and problematic mobile phone use among Chinese adolescents: A longitudinal moderated mediation model involving school engagement and sensation seeking’, *Child Abuse & Neglect*, vol. 115.

Chen, Y, Zhu, J & Zhang, W 2021, ‘Reciprocal longitudinal relations between peer victimization and mobile phone addiction: The explanatory mechanism of adolescent depression’, *Journal of Adolescence*, vol. 89, pp. 1-9.

Cui, G, Yin, Y, Li, S, Chen, L, Liu, X, Tang, K & Li, Y 2021, ‘Longitudinal relationships among problematic mobile phone use, bedtime procrastination, sleep quality and depressive symptoms in Chinese college students: a cross-lagged panel analysis’, *BMC Psychiatry*, vol. 21, no. 1.

Durak, HY 2019, ‘Investigation of nomophobia and smartphone addiction predictors among adolescents in Turkey: Demographic variables and academic performance’, *Social Science Journal*, vol. 56, no. 4, pp. 492-517.

Geng, J, Bao, L, Wang, H, Wang, J, Gao, T & Lei, L 2022, ‘Does childhood maltreatment increase the subsequent risk of problematic smartphone use among adolescents? A two-wave longitudinal study’, *Addictive Behaviors*, vol. 129.

Geng, J, Lei, L, Ouyang, M, Nie, J & Wang, P 2021, ‘The influence of perceived parental phubbing on adolescents’ problematic smartphone use: A two-wave multiple mediation model’, *Addictive Behaviors*, vol. 121.

Hong, W, Liu, R-D, Ding, Y, Oei, TP, Zhen, R & Jiang, S 2019, ‘Parents' Phubbing and Problematic Mobile Phone Use: The Roles of the Parent-Child Relationship and Children's Self-Esteem’, *Cyberpsychology Behavior and Social Networking*, vol. 22, no. 12, pp. 779-86.

Hong, W, Liu, RD, Ding, Y, Jiang, S, Yang, X & Sheng, X 2021, ‘Academic procrastination precedes problematic mobile phone use in Chinese adolescents: A longitudinal mediation model of distraction cognitions’, *Addictive Behaviors*, vol. 121.

Hong, W, Liu, RD, Ding, Y, Zhen, R, Jiang, R & Fu, X 2020, ‘Autonomy need dissatisfaction in daily life and problematic mobile phone use: The mediating roles of boredom proneness and mobile phone gaming’, *International Journal of Environmental Research and Public Health*, vol. 17, no. 15, pp. 1-13.

Hu, Z & Xiang, Y 2022, ‘Who Is the Chief Culprit, Loneliness, or Smartphone Addiction? Evidence from Longitudinal Study and Weekly Diary Method’, *International Journal of Mental Health and Addiction*.

Kang, Y, Liu, S, Yang, L, Xu, B, Lin, L, Xie, L, Zhang, W, Zhang, J & Zhang, B 2020, ‘Testing the bidirectional associations of mobile phone addiction behaviors with mental distress, sleep disturbances, and sleep patterns: A one-year prospective study among Chinese college students’, *Frontiers in Psychiatry*, vol. 11.

Lai, X, Huang, S, Nie, C, Yan, JJ, Li, Y, Wang, Y & Luo, Y 2022, ‘Trajectory of problematic smartphone use among adolescents aged 10-18 years: The roles of childhood family environment and concurrent parent-child relationships’, *Journal of Behavioral Addictions*, vol. 11, no. 2, pp. 577-87.

Li, Y, Lin, S, Yang, X, Sheng, J, Wang, L, Han, Y, Cao, Y & Chen, J 2023, ‘A Vicious Cycle: The Reciprocal Longitudinal Relationship Between Social Rejection, Social Avoidance, and Smartphone Addiction Among Adolescents’, *International Journal of Mental Health and Addiction*.

Shi, X, Wang, A & Zhu, Y 2023, ‘Longitudinal associations among smartphone addiction, loneliness, and depressive symptoms in college students: Disentangling between- And within-person associations’, *Addictive Behaviors*, vol. 142, p. 107676.

Wang, D, Nie, X, Zhang, D & Hu, Y 2022, ‘The relationship between parental psychological control and problematic smartphone use in early Chinese adolescence: A repeated-measures study at two time-points’, *Addictive Behaviors*, vol. 125.

Wang, X, Qiao, Y & Wang, S 2023, ‘Parental phubbing, problematic smartphone use, and adolescents' learning burnout: A cross-lagged panel analysis’, *Journal of Affective Disorders*, vol. 320, pp. 442-9.

Xie, J-Q, Zimmerman, MA, Rost, DH, Yin, X-Q & Wang, J-L 2020, ‘Stressful life events and problematic smartphone usage among Chinese boarding-school adolescents: A moderated mediation model of peer support and depressive symptoms’, *Addiction Research & Theory*, vol. 28, no. 6, pp. 493-500.

Yang, X, Liu, R-D, Ding, Y, Hong, W & Ding, Z 2022, ‘Interpersonal relationships moderate the relation between academic stress and mobile phone addiction via depression among Chinese adolescents: A three-wave longitudinal study’, *Current Psychology*.

Yuan, G, Elhai, JD & Hall, BJ 2021, ‘The influence of depressive symptoms and fear of missing out on severity of problematic smartphone use and internet gaming disorder among Chinese young adults: A three-wave mediation model’, *Addictive Behaviors*, vol. 112.

Zhang, K, Guo, H, Wang, T, Zhang, J, Yuan, G, Ren, J, Zhang, X, Yang, H, Lu, X, Zhu, Z, Du, J, Shi, H, Jin, G, Hao, J, Sun, Y, Su, P & Zhang, Z 2023, ‘A bidirectional association between smartphone addiction and depression among college students: A cross-lagged panel model’, *Frontiers in public health*, vol. 11, p. 1083856.

Zhang, Y, Li, S & Yu, G 2022, ‘The longitudinal relationship between boredom proneness and mobile phone addiction: Evidence from a cross-lagged model’, *Current Psychology*, vol. 41, no. 12, pp. 8821-8.

Zhao, C, Ding, N, Yang, X, Xu, H, Lai, X, Tu, X, Lv, Y, Xu, D & Zhang, G 2021, ‘Longitudinal Effects of Stressful Life Events on Problematic Smartphone Use and the Mediating Roles of Mental Health Problems in Chinese Undergraduate Students’, *Frontiers in public health*, vol. 9.

Zhou, H, Dang, L, Lam, LW, Zhang, MX & Wu, AMS 2021, ‘A cross-lagged panel model for testing the bidirectional relationship between depression and smartphone addiction and the influences of maladaptive metacognition on them in Chinese adolescents’, *Addictive Behaviors*, vol. 120.
