# Supplementary material for: Risk Factors of Smartphone Addiction: A Systematic Review of Longitudinal Studies
Source: Public Health Chall. 2024 Jun 22;3(2):e202. doi: 10.1002/puh2.202 (PMC12039634; doi:10.1002/puh2.202)
Supplement: Supplementary file 3 — Supporting Information [file PUH2-3-e202-s003.docx]

Supplementary Table 3 - Summary table of included and excluded articles longitudinal studies.

| Reference | Country | Sample size | Duration | SA assessment tool | Outcomes | Full text availability | Comment |  |
| --- | --- | --- | --- | --- | --- | --- | --- | --- |
| (Bae 2015) | South Korea | 2218 | Korean children and youth panel survey of the National Youth Policy Institute. | [Lee, Kim, and Na (2002)](https://journals-sagepub-com.ezproxy.uow.edu.au/doi/full/10.1177/0143034315604017#bibr28-0143034315604017) | - higher democratic was related to lower addictive use of smartphones - friendship satisfaction and academic motivation negatively influenced the addictive use of smartphones - supervisory parenting can positively impact peer relationship and learning motivation, and the satisfaction of peer relationship and academic motivation can be protective factors for the addictive use of smartphones | Yes | Methodology not in line with SR:  Secondary data | Reject |
| (Chen, Zhang, et al. 2021) | China | 1987 adolescents | 3 waves  1 year | ([Leung, 2008a](https://www-sciencedirect-com.ezproxy.uow.edu.au/science/article/pii/S0145213421000648#bib0150),[2008b](https://www-sciencedirect-com.ezproxy.uow.edu.au/science/article/pii/S0145213421000648#bib0155)) | - retrospective reports of childhood emotional neglect were positively associated with adolescent PMPU and school engagement fully mediated the association. - sensation seeking at Wave 3 moderated the pathway from school engagement to later adolescent PMPU in the mediated model. | Yes |  |  |
| (Chen, Zhu, et al. 2021) | China | 1820  Adolescents | 3 Waves | (Leung, 2008) | - Peer victimization at W1/W2 predicted MPA at W2/W3, and the inverse relation was also significant: MPA at W1/W2 positively predicted peer victimization at W2/W3 - the bidirectional association between peer victimization and MPA was mediated by adolescent depression | Yes |  |  |
| (Chiang et al. 2019) | Taiwan | 2155 children | 1 year | (Lin et al. 2017) | - Both boys and girls tended to transition to tolerance or high- smartphone addiction proneness statuses - children’s depression and their usage of social networking sites increased the risk of smartphone addiction. | yes | Poorly written | Reject |
| (Choe & Yu 2022a) |  | 1737 adolescents | 3 years | Korean Children and Youth Panel Survey | - Friendships reduce mobile phone dependency (MPD), depressive symptoms increase MPD | Not available | Methodology not in line with SR:  Secondary data  Full text not available | Reject |
| (Choe & Yu 2022b) | Korea | 1659 adolescents | Korean Children and Youth Panel Survey | [Lee, Kim, and Na (2002)](https://journals-sagepub-com.ezproxy.uow.edu.au/doi/full/10.1177/0143034315604017#bibr28-0143034315604017) | - Superior school adaptation decreased mobile phone dependence, but mobile phone dependence did not affect school adaptation. - Girls were found to have a higher level of mobile phone dependence than boys did. - Child abuse and neglect played a role in increasing mobile phone dependence and negatively affected school adaptation | Yes | Methodology not in line with SR:  Secondary data | Reject |
| (Chun et al. 2022) | Korea | 360 adolescents | Case control study before and after covid-19 |  | - personal-level factors (i.e., gender, depressive symptoms, self-control, and cyberbullying victimization) are associated with smartphone addiction - For the group whose smartphone usage time increased after the outbreak, economic status, academic performance, and academic year were related to smartphone addiction. | Yes | Methodology not in line with SR:  Cross-sectional | Reject |
| (Coyne et al. 2019) | USA | 385 | 3 years | (Merlo et al. 2013) | - problematic cell phone use predicted later depression but depression did not predict PSU - Self-regulation and problematic cell phone use are not longitudinally related. - Early problematic cell phone use is moderately associated with later depression | Yes | Outcome not in line with SR | Reject |
| (Cui et al. 2021) | China | 1181 | 2 years | (Xiong et al., 2012) | - significant bidirectional relationships of problematic mobile phone use with bedtime procrastination and depressive symptoms - there were also significant bidirectional relationships of sleep quality with bedtime procrastination and depressive symptoms | Yes |  |  |
| (Dey et al. 2019) | Switzerland | 5,096 | 3 waves | (Kwon et al. 2013) |  | yes | Methodology not in line with SR:  Did NOT evaluate longitudinal data | Reject |
| (Durak 2019) | Turkey | 612 adolescents | 2 waves  1 year | (Kwon et al. 2013) | - age, educational level and gender variables predict smartphone addiction significantly - internet usage experience and daily internet usage time variables are significant predictors of smartphone use disorder - Smartphone control frequency Smartphone usage experience Smartphone usage purpose are predictors of SA | yes | Methodology not in line with SR:  Cross-sectional | Reject |
| (Elhai et al. 2018) | USA | 261 | 2 waves  1 month | (Kwon et al. 2013) | - Distress tolerance inversely predicted problematic smartphone use (PSU). - Mindfulness inversely predicted PSU. - Mindfulness mediated relations between depression and PSU severity. - Mindfulness mediated relations between anxiety sensitivity and PSU severity. - Distress tolerance mediated relations between anxiety sensitivity and PSU severity. | yes | Methodology not in line with SR:  Study period < 6month | Reject |
| (Ferrara et al. 2020) | Italy | 100 | 8 month | Unclear | - persistent smartphone utilization is a risk factor for children's health | No | Outcomes not in line with/ relevant to SR | Reject |
| (Geng et al. 2022) | China | 890 adolescents | 2 waves  6 month | [Csibi, Demetrovics, and Szabo (2016)](https://www-sciencedirect-com.ezproxy.uow.edu.au/science/article/pii/S0306460322000168#b0055) | - Childhood maltreatment experiences predicted adolescents’ problematic smartphone use. | yes |  |  |
| (Geng et al. 2021) | China | 1447 | 2 waves  6 month | (Kwon et al. 2013) | - Perceived parental phubbing (PP) predicted adolescents’ subsequent problematic smartphone use (PSU). - Loneliness mediated the link between PP and adolescents’ subsequent PSU. - Loneliness and fear of missing out sequentially mediated the link between PP and adolescents’ subsequent PSU. | yes |  |  |
| (Ghasempour & Mahmoodi-Aghdam 2015) |  |  | Not longitudinal |  |  | yes | Methodology not in line with SR:  Cross-sectional | Reject |
| (Herrero et al. 2019) |  | 241 | 3 years | SAPS; Bian & Leung, 2015) | - the more addiction decreases, the more social support increases - high levels of addiction and relatively low levels of support remained stable over time in a group of users | yes | Outcomes not in line/ not relevant to SR | Reject |
| (Herrero et al. 2021) |  | 716 | 3 years | SAPS; Bian & Leung, 2015 | - the probability of cyberfraud victimization is lower among users with a decrease in smartphone addiction and an increase in social support | Yes | Outcomes not in line/ not relevant to SR | Reject |
| (Hong et al. 2021) | China | 633  Adolescents | 3 waves  1.5 years | MPPUS-10)  (Foerster et al. 2015) | - academic procrastination positively predicted subsequent PMPU, but the reverse prediction was not stable - distraction cognitions played a mediating role in linking earlier academic procrastination and subsequent PMPU | Yes |  |  |
| (Hong et al. 2020) | China | 358 adolescents | 3 waves  2 years | (Foerster et al. 2015) | - autonomy need dissatisfaction not only directly predicted PMPU, but also exerted effects via the mediating role of boredom proneness and the chain mediating role of boredom proneness and mobile phone gaming | Yes |  |  |
| (Hong et al. 2019) | China | 1721 adolescents | 2 waves  6 month | Hong et al., 2019 | - parental phubbing predicts subesqunet PSU - the parent–child relationship mediates the relationship between parental phubbing predicts subesqunet PSU | yes |  |  |
| (Hu et al. 2022) | China | 579  University students | 3 waves  6 month | [Su et al. (2014)](https://www-sciencedirect-com.ezproxy.uow.edu.au/science/article/pii/S0747563221003812#bib96) | - Interpersonal alienation and meaning in life increased with the epidemic under control. - The risk of smartphone addiction decreased with the epidemic under control. - Interpersonal alienation negatively predicted subsequent meaning in life. - Interpersonal alienation positively predicted subsequent smartphone addiction - Meaning in life had indirect effects on interpersonal alienation and smartphone addiction. | Yes | Outcomes not in line/ not relevant to SR:  Focus on changes in SA over time. | Reject |
| (Hu & Xiang 2022) | China | 906 adolescents | 1 year | Csibi, S., Demetrovics, Z., & Szabo, A. (2016) | - trait loneliness positively predicted smartphone addiction among adolescents - smartphone addiction could not predict loneliness | Yes |  |  |
| (Jahrami et al. 2022) |  | 306 | 2 waves | NMP-Q  (Yildirim, Correia, 2015) | - symptoms of nomophobia increased significantly during a social media outage - Baseline insomnia scores predicted a surge in the global scores of nomophobia symptoms during a social media outage. | Yes | Outcomes not in line/ not relevant to SR | Reject |
| (Jeong et al. 2022) | Korea | 3615 pairs of children and their mothers |  | Korean Children and Youth Panel Survey | - mothers and children’s smartphone addiction significantly increased over time - mother’s smartphone addiction was found to have a significant effect on the child's initial value and the change rate. Moreover, children's smartphone addiction change rate was significantly affected by the change rate of the mother's smartphone addiction | yes | Outcomes not in line/ not relevant to SR | Reject |
| (Jun 2016) | Korea | 1877 children | 3 Years | Korean Children and Youth Panel Survey | - SA affected depressive symptoms over time. - Depressive symptoms influenced mobile phone addiction over time. - Bidirectional relationship between depressive symptoms and SA | Yes | Methodology not in line with SR:  Secondary data | Reject |
| (Kang et al. 2020) | China | 902 students | 1 year | (Walsh et al. 2011) | - the severity of depression and anxiety at baseline were predictive factors of MPAB at one-year follow-up, but not in the opposite direction | yes |  |  |
| (Khoo & Yang 2021) | Singapore | 217 | 5 weeks | Measured smartphone coping and use rather than SA | - Mental disengagement fully mediated the relation between rumination and the initial level (intercept) of smartphone use. - We found that an objective measure of smartphone use is more valid than a self-report measure. | Yes | Methodology not in line with SR:  Study period < 6month  Inadequate SA measures | Reject |
| (Kim et al. 2020) | Korea | 2541 |  | Korean Children and Youth Panel Survey 2018 (KCYPS 2018) | - both maternal smartphone dependency and negative parenting were determinants of adolescent smartphone dependency | yes | Methodology not in line with SR:  Secondary data | Reject |
| (Kim & Lee 2022) | Korea | 53,534 |  | Korea Youth Risk Behavior Web-based Survey | - Participating in regular physical activity is effective in preventing SA | Yes | Methodology not in line with SR:  Secondary data  Outcome not relevant to SR | Reject |
| (Kim 2019) |  | 288 | 2 waves  4 month | Bianchi & Phillips, 2005; Kwon, Kim, Cho, & Yang, 2013 | - loneliness leads to problematic use of smartphones, which reduces face-to-face interaction while increasing need for social assurance. - However, need for social assurance fueled by excessive smartphone use is usually not gratified, and eventually leads to greater loneliness. |  | Methodology not in line with SR:  Study period < 6month | Reject |
| (Kuem et al. 2021) |  | 441adults | 2 waves  4 weeks apart | Adapted their own measures from existing | - study significantly contributes to the information systems literature by offering a refined model of smartphone addiction that effectively describes pathological smartphone use while correcting critical biases in existing models. | yes | Methodology not in line with SR:  Study period < 6month | Reject |
| (Lai et al. 2022) | China | 2,548 adolescents and their parents | 3 waves  3 years | (Kim et al. 2014) | - Early life socioeconomic status, childhood family unpredictability, and the concurrent parent-child relationship had unique impacts on the development of problematic smartphone use during adolescence. | Yes |  |  |
| (Lapierre et al. 2019) | USA | 346 adolescents | 2 waves  3 month | (Kim et al. 2014) | - smartphone dependency predicted loneliness and depressive symptoms - loneliness predicted depressive symptoms - smartphone use predicted smartphone dependency | yes | Methodology not in line with SR:  Study period < 6month | Reject |
| (Li et al. 2023) | China | 1368 adolescents | 3 waves  1 year | Unclear | - reciprocal relationship between social rejection and smartphone addiction, with social avoidance playing a mediating role | yes |  |  |
| (Lim & You 2019) | Korea | 485 elementary school students | 2 years | National Youth Policy Institute (NYPI) Survey | - parental negligence had a statistically significant positive effect on all subfactors of peer attachment - communication and trust were not significantly related to mobile phone dependency, - isolation was related to mobile phone dependency - while perceived parental negligence did not have a direct effect on mobile phone dependency, it did have an indirect effect via isolation | yes | Methodology not in line with SR:  Secondary data | Reject |
| (Noë et al. 2019) | UK | 64 | 8 weeks | SAS  (Kwon et al. 2013) | - Lifestyle apps are associated with Smartphone Addiction, especially for female users. - Social apps in general are associated with Smartphone Addiction. - User interactions with Snapchat strongly correlate with Smartphone Addiction. | yes | Methodology not in line with SR:  Study period < 6month | Reject |
| (Park et al. 2019) | Korea | 1794 adolescents | 4 waves | [Lee, Kim, and Na (2002)](https://journals-sagepub-com.ezproxy.uow.edu.au/doi/full/10.1177/0143034315604017#bibr28-0143034315604017)  KCYPS | - Bidirectionally relationship for depression and smartphone dependence - No gender difference found - No reciprocal causal relationship between smartphone use and dependence - Reciprocal causal relationship for depression and smartphone dependence at T2 to T3 (but not for T1 to T2) | yes | Methodology not in line with SR:  Secondary data | Reject |
| (Poulain et al. 2021) | Germany | 363 adolescents | 2 waves  1 year | (Kim et al. 2014) | - Children who reported prolonged periods of smartphone use or more symptoms of smartphone addiction exhibited significantly higher levels of externalizing and internalizing behavioral difficulties at t1 and t2. | Yes | Outcomes not in line/ not relevant to SR | Reject |
| (Qiu, Li, et al. 2022) | China | 527 adolescents | 3 waves  1 year | (Leung 2008) | - Parent-child relationship was negatively associated with subsequent SA - Hope mediated the link between parent–child relationship and SA. - The impact of parent–child relationship on hope was strengthened with increase in life satisfaction. - The indirect link between parent–child relationship and SA via hope was stronger for adolescents with high level of life satisfaction than those with low level of life satisfaction. | Yes | Outcomes not in line with SR:  Protective factor, rather then predictive factor | Reject |
| (Qiu, Liu, et al. 2022) | China | 478 children and adolescents | 3 waves  1 year | (Leung 2008) | - Presence of meaning and search for meaning are negatively associated with problematic smartphone use among children and adolescents. - Depression and self-control mediated the link between presence of meaning and subsequent problematic smartphone use respectively. - Depression and self-control sequentially mediated the link between presence of meaning and subsequent problematic smartphone use, whereas not sequentially mediated the relationship between search for meaning and subsequent problematic smartphone use. | yes | Methodology not in line with SR:  Definition of presence of meaning is inconclusive, thus difficult to make a conclusion. | Reject |
| (Rozgonjuk et al. 2019) |  | 261 college students | 2 waves  1 month | (Kwon et al. 2013) | - intolerance of uncertainty (IU) was related to non-social smartphone use and PSU. - Non-social smartphone use mediated relations between IU and PSU. | Yes | Methodology not in line with SR:  Study period < 6month | Reject |
| (Rozgonjuk et al. 2018) |  | 101 uni students | 1 week | (Kwon et al. 2013) | - PSU positively predicted screentime minutes (but not screen unlocks) over a week. - Depression negatively predicted screen unlocks (but not screentime) over a week. | yes | Methodology not in line with SR:  Study period < 6month | Reject |
| (Serra et al. 2021) |  | 185 children adolescents | 2 month? | De Pasquale, Sciacca & Hichy  2017 | - High risk of SA and SA increased during COVID-19 compared to pre-pandemic | yes | Methodology not in line with SR:  Study period < 6month | Reject |
| (Shi et al. 2023) | China | 3,827 college students | 4 waves  2 years | (Kwon et al. 2013) | - [Smartphone addiction](https://www-sciencedirect-com.ezproxy.uow.edu.au/topics/medicine-and-dentistry/computer-addiction) and depressive symptoms were bidirectionally related. - Loneliness mediated the association between smartphone addiction and depressive symptoms at the within-person level. - Gender differences were not found in these relationships. | yes |  |  |
| (Tossell et al. 2015) | USA | 34 university students | 1 year | (Koo, 2009)  (Young, 1998) | - Participants did not own a smartphone before and were given one. - Participants who got addicted to the smartphone used device twice as much and launched applications much more frequently as compared to the non-addicted user, these include Mail, Messaging, Facebook and the Web | yes | Methodology not in line with SR:  Small sample size  Outcomes not relevant to SR | Reject |
| (Wang et al. 2022) | China | 2128 4th and 5th grade students | 2 waves  1 year | (Foerster et al. 2015) | - reciprocal relationship between parental psychological control and PSU severity in early adolescence - Both psychological security and insomnia mediate the link between parental psychological control and subsequent PSU severity. - Psychological security and insomnia play serial mediating roles between parental psychological control and subsequent PSU severity | yes |  |  |
| (Wang et al. 2023) | China | 2260 adolescents | 2 waves  1 year | (Wang et al., 2019) | - Parental phubbing was associated with PSU - bidirectional associations between PSU and learning burnout as well as between parental phubbing and learning burnout. - PSU significantly mediated the relationship between parental phubbing and learning burnout. - There were no gender differences among parental phubbing, problematic smartphone use, and learning burnout. | yes |  |  |
| (Wu & Liu 2023) | China | 445 college students | 4 waves  6 month | (Su et al., 2014) | - During COVID-19 - Social support and family functioning were protective factors for depressive symptoms. - Higher risk perception and [smartphone addiction](https://www-sciencedirect-com.ezproxy.uow.edu.au/topics/medicine-and-dentistry/computer-addiction) can increase depressive symptoms. - Depressive symptoms positively predicted smartphone addiction and negatively predicted state hope. | yes | Outcomes not in line with SR:  Inadequate data on how covid-19 influences SA | Reject |
| (Xie et al. 2020) | China | 124 adolescents | 3 waves  2 years | Huang et al. [Citation2014](https://www-tandfonline-com.ezproxy.uow.edu.au/doi/full/10.1080/16066359.2019.1692824) | - Depressive symptoms were positively correlated with PSU - males’ PSU was significantly higher than females | yes |  |  |
| (Yang et al. 2021) | China | 195 university students | 4 waves  2 years | Huang et al. [Citation2014](https://www-tandfonline-com.ezproxy.uow.edu.au/doi/full/10.1080/16066359.2019.1692824)  (Leung 2008) | - 3 waves before COVID-19, one after lockdowns - significant increase in the levels of depressive symptoms and prevalence of probable depression during COVID-19 compared to those 18 months, 12 months and 6 months before COVID-19 but non-significant changes in smart phone addiction (SPA) - Boredom and emotional loneliness were positively associated with both SPA and depressive symptoms during COVID-19. - Social loneliness was also positively associated with depressive symptoms during COVID-19. - Quarantine and lockdown were not significantly associated with SPA or depressive symptoms. | yes | Outcomes not in line with SR:  Data about COVID-19 and SA is cross-sectional | Reject |
| (Yang, X et al. 2022) | China | 642 adolescents | 4 waves  1.5 years | (Foerster et al. 2015) | - academic stress positively predicted PMPU after controlling for the effects of gender and grade - academic stress positively predicted depression which in turn positively predicted PMPU - depression partially mediated the relation between academic stress and PMPU - academic stress and interpersonal relationships negatively predicted depression which in turn predicted PMPU | yes |  |  |
| (Yang, Y et al. 2022) | China | 258 primary school students | 3 waves  1 year | (Foerster et al. 2015) | - Parent-child relationships negatively predict subsequent PMPU, and PMPU negatively predicts subsequent parent-child relationships. - children with poor parent-child relationships are more likely to engage in PMPU | yes | Results not in line with outcomes for SR:  Protective factor rather than predictive factor | Reject |
| (Yoo et al. 2022) | South Korea | 184 children and adolescents | 3 waves  9 month | (Kwon et al. 2013)  (Kim et al. 2014) | - Harm avoidance (HA) displayed robust positive correlations with PSU at all time points. | yes | Methodology not in line with SR:  Study population not clearly defined, recruited from a larger cohort study | Reject |
| (Yuan et al. 2021) | China | 341 university students | 3 waves  1 year | (Kwon et al. 2013) | - depression severity was significantly related to increased symptoms of PSU and IGD symptoms. - FoMO significantly mediated the linkage between depression and PSU severity. - IGD symptoms also partially mediated the relation between FoMO and PSU severity | yes |  |  |
| (Yun et al. 2022) | Korea | 342 children |  | Panel Study on Korean Children | - Risk factors for PSU are child’s externalizing problems and permissive parenting behaviour - Protective factors for PSU are peer communication, parental supervision, and authoritative parenting behaviour | yes | Methodology not in line with SR:  Secondary data | Reject |
| (Zhang et al. 2020) | China | 265 university students | 3 waves 3 years | MPATS; [Xiong et al., 2012](https://www-sciencedirect-com.ezproxy.uow.edu.au/science/article/pii/S016503271930775X#bib0060) | - mobile phone dependence at Year 1 significantly predicted poor mental health status at Year 3 - Did not measure mental health at baseline | yes | Outcomes not in line/ relevant to SR:  tested direction of health outcome | Reject |
| (Zhang et al. 2023) | China | 1,186 adolescents | 2 waves 1 year | (Kwon et al. 2013) | - significant bidirectional association between smartphone addiction and depression among freshmen, but only in the female population - In contrast, the cross-lagged model in the male group showed no predictive effect between SA and depression | yes |  |  |
| (Zhang et al. 2022) | China | 352 college students | 2 waves  8 month | Xiong et al., [2012](https://link-springer-com.ezproxy.uow.edu.au/article/10.1007/s12144-020-01333-8#ref-CR36) | - boredom proneness and mobile phone addiction are mutually influenced, and - mobile phone addiction is a stronger predictor of boredom proneness than the other way around - the bidirectional relationship remained consistent across gender | yes |  |  |
| (Zhao et al. 2021) | China | 197 university students | 3 waves  1 year | (Leung 2008) | - stressful life events and mental health problems (i.e., depressive symptoms, poor sleep quality, and suicidal ideation) are predictors of problematic smartphone use | yes |  |  |
| (Zhou et al. 2021) | China | 313 high school students | 2 waves 6 month | (Lin et al. 2014) | - Depression was a salient predictor for [smartphone addiction](https://www-sciencedirect-com.ezproxy.uow.edu.au/topics/medicine-and-dentistry/computer-addiction). - Smartphone addiction was not a salient predictor for depression. - Maladaptive metacognition was positively correlated with depression and smartphone addiction at both waves. - Maladaptive metacognition predicted depression but not smartphone addiction 6 months later. - Maladaptive metacognition had an indirect effect on smartphone addiction via depression. | yes |  |  |
| (Zwilling 2022) | Israel | 197 general public | Two questionaries | Hong et al., 2012 | - Questionnaires administer at the same time, but referring to pre and post Covid lockdowns. - nomophobia, social affiliation, and sleep hours affect PSU. - the indirect effect of the number of sleep hours on stress and PSU was found to be significant in T2 and in T1 | yes | Methodology not in line with SR:  Cross-sectional | Reject |

Bae, SM 2015, ‘The relationships between perceived parenting style, learning motivation, friendship satisfaction, and the addictive use of smartphones with elementary school students of South Korea: Using multivariate latent growth modeling’, *School Psychology International*, vol. 36, no. 5, pp. 513-31.

Chen, Y, Zhang, Y, Zhang, L, Luo, F, Xu, W, Huang, J, Yang, L & Zhang, W 2021, ‘Childhood emotional neglect and problematic mobile phone use among Chinese adolescents: A longitudinal moderated mediation model involving school engagement and sensation seeking’, *Child Abuse & Neglect*, vol. 115.

Chen, Y, Zhu, J & Zhang, W 2021, ‘Reciprocal longitudinal relations between peer victimization and mobile phone addiction: The explanatory mechanism of adolescent depression’, *Journal of Adolescence*, vol. 89, pp. 1-9.

Chiang, J-T, Chang, F-C, Lee, K-W & Hsu, S-Y 2019, ‘Transitions in smartphone addiction proneness among children: The effect of gender and use patterns’, *PLoS ONE*, vol. 14, no. 5.

Choe, C & Yu, S 2022a, ‘Longitudinal Cross-Lagged Analysis Between Mobile Phone Dependence, Friendships, and Depressive Symptoms Among Korean Adolescents’, *Cyberpsychology, behavior and social networking*, vol. 25, no. 7, pp. 450-7.

—— 2022b, ‘The reciprocal relationship between mobile phone dependence and school adaptation in Korean adolescents: Autoregressive cross-lagged modeling’, *Acta psychologica*, vol. 228, p. 103628.

Chun, J, Lee, HK, Jeon, H, Kim, J & Lee, S 2022, ‘Impact of COVID-19 on Adolescents' Smartphone Addiction in South Korea’, *Social work in public health*.

Coyne, SM, Stockdale, L & Summers, K 2019, ‘Problematic cell phone use, depression, anxiety, and self-regulation: Evidence from a three year longitudinal study from adolescence to emerging adulthood’, *Computers in Human Behavior*, vol. 96, pp. 78-84.

Cui, G, Yin, Y, Li, S, Chen, L, Liu, X, Tang, K & Li, Y 2021, ‘Longitudinal relationships among problematic mobile phone use, bedtime procrastination, sleep quality and depressive symptoms in Chinese college students: a cross-lagged panel analysis’, *BMC Psychiatry*, vol. 21, no. 1.

Dey, M, Studer, J, Schaub, MP, Gmel, G, Ebert, DD, Lee, JY-C & Haug, S 2019, ‘Problematic smartphone use in young Swiss men: Its association with problematic substance use and risk factors derived from the pathway model’, *Journal of Behavioral Addictions*, vol. 8, no. 2, pp. 326-34.

Durak, HY 2019, ‘Investigation of nomophobia and smartphone addiction predictors among adolescents in Turkey: Demographic variables and academic performance’, *Social Science Journal*, vol. 56, no. 4, pp. 492-517.

Elhai, JD, Levine, JC, O'Brien, KD & Armour, C 2018, ‘Distress tolerance and mindfulness mediate relations between depression and anxiety sensitivity with problematic smartphone use’, *Computers in Human Behavior*, vol. 84, pp. 477-84.

Ferrara, P, Infantino, C, Franceschini, G & Sacco, R 2020, ‘Smartphone and tablet addiction among children: A significant predictor of behavioral and physical alterations’, *Minerva Psichiatrica*, vol. 61, no. 1, pp. 20-4.

Foerster, M, Roser, K, Schoeni, A & Röösli, M 2015, ‘Problematic mobile phone use in adolescents: derivation of a short scale MPPUS-10’, *International journal of public health*, vol. 60, no. 2, pp. 277-86.

Geng, J, Bao, L, Wang, H, Wang, J, Gao, T & Lei, L 2022, ‘Does childhood maltreatment increase the subsequent risk of problematic smartphone use among adolescents? A two-wave longitudinal study’, *Addictive Behaviors*, vol. 129.

Geng, J, Lei, L, Ouyang, M, Nie, J & Wang, P 2021, ‘The influence of perceived parental phubbing on adolescents’ problematic smartphone use: A two-wave multiple mediation model’, *Addictive Behaviors*, vol. 121.

Ghasempour, A & Mahmoodi-Aghdam, M 2015, ‘The Role of Depression and Attachment Styles in Predicting Students' Addiction to Cell Phones’, *Addiction & health*, vol. 7, no. 3-4, pp. 192-7.

Herrero, J, Torres, A, Vivas, P & Urueña, A 2019, ‘Smartphone Addiction and Social Support: A Three-year Longitudinal Study’, *Intervención Psicosocial*, vol. 28, no. 3, pp. 111-8.

Herrero, J, Torres, A, Vivas, P & Urueña, A 2021, ‘Smartphone Addiction, Social Support, and Cybercrime Victimization: A Discrete Survival and Growth Mixture Model’, *Psychosocial Intervention*, vol. 31, no. 1, pp. 59-66.

Hong, W, Liu, R-D, Ding, Y, Oei, TP, Zhen, R & Jiang, S 2019, ‘Parents' Phubbing and Problematic Mobile Phone Use: The Roles of the Parent-Child Relationship and Children's Self-Esteem’, *Cyberpsychology Behavior and Social Networking*, vol. 22, no. 12, pp. 779-86.

Hong, W, Liu, RD, Ding, Y, Jiang, S, Yang, X & Sheng, X 2021, ‘Academic procrastination precedes problematic mobile phone use in Chinese adolescents: A longitudinal mediation model of distraction cognitions’, *Addictive Behaviors*, vol. 121.

Hong, W, Liu, RD, Ding, Y, Zhen, R, Jiang, R & Fu, X 2020, ‘Autonomy need dissatisfaction in daily life and problematic mobile phone use: The mediating roles of boredom proneness and mobile phone gaming’, *International Journal of Environmental Research and Public Health*, vol. 17, no. 15, pp. 1-13.

Hu, Q, Liu, Q & Wang, Z 2022, ‘Meaning in life as a mediator between interpersonal alienation and smartphone addiction in the context of covid-19: A three-wave longitudinal study’, *Computers in Human Behavior*, vol. 127.

Hu, Z & Xiang, Y 2022, ‘Who Is the Chief Culprit, Loneliness, or Smartphone Addiction? Evidence from Longitudinal Study and Weekly Diary Method’, *International Journal of Mental Health and Addiction*.

Jahrami, H, Fekih-Romdhane, F, Saif, Z, Bragazzi, NL, Pandi-Perumal, SR, BaHammam, AS & Vitiello, MV 2022, ‘A Social Media Outage Was Associated with a Surge in Nomophobia, and the Magnitude of Change in Nomophobia during the Outage Was Associated with Baseline Insomnia’, *Clocks & sleep*, vol. 4, no. 4, pp. 508-19.

Jeong, K-H, Kim, S, Ryu, JH & Lee, S 2022, ‘A Longitudinal Relationship Between Mother's Smartphone Addiction to Child's Smartphone Addiction’, *International Journal of Mental Health and Addiction*, pp. 1-12.

Jun, S 2016, ‘The reciprocal longitudinal relationships between mobile phone addiction and depressive symptoms among Korean adolescents’, *Computers in Human Behavior*, vol. 58, pp. 179-86.

Kang, Y, Liu, S, Yang, L, Xu, B, Lin, L, Xie, L, Zhang, W, Zhang, J & Zhang, B 2020, ‘Testing the bidirectional associations of mobile phone addiction behaviors with mental distress, sleep disturbances, and sleep patterns: A one-year prospective study among Chinese college students’, *Frontiers in Psychiatry*, vol. 11.

Khoo, SS & Yang, H 2021, ‘Mental disengagement mediates the effect of rumination on smartphone use: A latent growth curve analysis’, *Computers in Human Behavior*, vol. 120.

Kim, C, Kang, KI & Lee, N 2020, ‘Intergenerational transmissions of mother–adolescent smartphone dependency: The mediating role of negative parenting and the moderating role of gender’, *International Journal of Environmental Research and Public Health*, vol. 17, no. 16, pp. 1-13.

Kim, D, Lee, Y, Lee, J, Nam, JK & Chung, Y 2014, ‘Development of Korean Smartphone addiction proneness scale for youth’, *PLoS ONE*, vol. 9, no. 5, pp. e97920-e.

Kim, J-H 2019, ‘Longitudinal associations among psychological issues and problematic use of smartphones: A two-wave cross-lagged study’, *Journal of Media Psychology: Theories, Methods, and Applications*, vol. 31, no. 3, pp. 117-27.

Kim, J & Lee, K 2022, ‘The Association between Physical Activity and Smartphone Addiction in Korean Adolescents: The 16th Korea Youth Risk Behavior Web-Based Survey, 2020’, *Healthcare (Basel, Switzerland)*, vol. 10, no. 4.

Kuem, J, Ray, S, Hsu, P-F & Khansa, L 2021, ‘Smartphone Addiction and Conflict: An Incentive-Sensitisation Perspective of Addiction for Information Systems’, *European Journal of Information Systems*, vol. 30, no. 4, pp. 403-24.

Kwon, M, Dai-Jin, K, Cho, H & Yang, S 2013, ‘The Smartphone Addiction Scale: Development and Validation of a Short Version for Adolescents’, *PLoS ONE*, vol. 8, no. 12.

Lai, X, Huang, S, Nie, C, Yan, JJ, Li, Y, Wang, Y & Luo, Y 2022, ‘Trajectory of problematic smartphone use among adolescents aged 10-18 years: The roles of childhood family environment and concurrent parent-child relationships’, *Journal of Behavioral Addictions*, vol. 11, no. 2, pp. 577-87.

Lapierre, MA, Zhao, P & Custer, BE 2019, ‘Short-term longitudinal relationships between smartphone use/dependency and psychological well-being among late adolescents’, *Journal of Adolescent Health*, vol. 65, no. 5, pp. 607-12.

Leung, L 2008, ‘LINKING PSYCHOLOGICAL ATTRIBUTES TO ADDICTION AND IMPROPER USE OF THE MOBILE PHONE AMONG ADOLESCENTS IN HONG KONG’, *Journal of children and media*, vol. 2, no. 2, pp. 93-113.

Li, Y, Lin, S, Yang, X, Sheng, J, Wang, L, Han, Y, Cao, Y & Chen, J 2023, ‘A Vicious Cycle: The Reciprocal Longitudinal Relationship Between Social Rejection, Social Avoidance, and Smartphone Addiction Among Adolescents’, *International Journal of Mental Health and Addiction*.

Lim, SA & You, S 2019, ‘Effect of parental negligence on mobile phone dependency among vulnerable social groups: Mediating effect of peer attachment’, *Psychological Reports*, vol. 122, no. 6, pp. 2050-62.

Lin, Y-H, Chang, L-R, Lee, Y-H, Tseng, H-W, Kuo, TBJ & Chen, S-H 2014, ‘Development and validation of the Smartphone Addiction Inventory (SPAI)’, *PLoS ONE*, vol. 9, no. 6, pp. e98312-e.

Lin, YH, Pan, YC, Lin, SH & Chen, SH 2017, ‘Development of short‐form and screening cutoff point of the Smartphone Addiction Inventory (SPAI‐SF)’, *International Journal of Methods in Psychiatric Research*, vol. 26, no. 2, pp. e1525-n/a.

Merlo, LJ, Stone, AM & Bibbey, A 2013, ‘Measuring Problematic Mobile Phone Use: Development and Preliminary Psychometric Properties of the PUMP Scale’, *Journal of addiction*, vol. 2013, p. 912807.

Noë, B, Turner, LD, Linden, DEJ, Allen, SM, Winkens, B & Whitaker, RM 2019, ‘Identifying indicators of smartphone addiction through user-app interaction’, *Computers in Human Behavior*, vol. 99, pp. 56-65.

Park, S-Y, Yang, S, Shin, C-S, Jang, H & Park, S-Y 2019, ‘Long-Term Symptoms of Mobile Phone Use on Mobile Phone Addiction and Depression Among Korean Adolescents’, *International Journal of Environmental Research and Public Health*, vol. 16, no. 19.

Poulain, T, Vogel, M, Kliesener, T & Kiess, W 2021, ‘Associations between changes in behavioral difficulties and levels of problematic smartphone use in adolescents over a 1-year period’, *European child & adolescent psychiatry*.

Qiu, C, Li, R, Luo, H, Li, S & Nie, Y 2022, ‘Parent-child relationship and smartphone addiction among Chinese adolescents: A longitudinal moderated mediation model’, *Addictive Behaviors*, vol. 130, p. 107304.

Qiu, C, Liu, Q, Yu, C, Li, Z & Nie, Y 2022, ‘The influence of meaning in life on children and adolescents’ problematic smartphone use: A three-wave multiple mediation model’, *Addictive Behaviors*, vol. 126.

Rozgonjuk, D, Elhai, JD, Täht, K, Vassil, K, Levine, JC & Asmundson, GJG 2019, ‘Non-social smartphone use mediates the relationship between intolerance of uncertainty and problematic smartphone use: Evidence from a repeated-measures study’, *Computers in Human Behavior*, vol. 96, pp. 56-62.

Rozgonjuk, D, Levine, JC, Hall, BJ & Elhai, JD 2018, ‘The association between problematic smartphone use, depression and anxiety symptom severity, and objectively measured smartphone use over one week’, *Computers in Human Behavior*, vol. 87, pp. 10-7.

Serra, G, Lo Scalzo, L, Giuffrè, M, Ferrara, P & Corsello, G 2021, ‘Smartphone use and addiction during the coronavirus disease 2019 (COVID-19) pandemic: cohort study on 184 Italian children and adolescents’, *Italian journal of pediatrics*, vol. 47, no. 1.

Shi, X, Wang, A & Zhu, Y 2023, ‘Longitudinal associations among smartphone addiction, loneliness, and depressive symptoms in college students: Disentangling between- And within-person associations’, *Addictive Behaviors*, vol. 142, p. 107676.

Tossell, C, Kortum, P, Shepard, C, Rahmati, A & Zhong, L 2015, ‘Exploring smartphone addiction: Insights from long-term telemetric behavioral measures’, *International Journal of Interactive Mobile Technologies*, vol. 9, no. 2, pp. 37-43.

Wang, D, Nie, X, Zhang, D & Hu, Y 2022, ‘The relationship between parental psychological control and problematic smartphone use in early Chinese adolescence: A repeated-measures study at two time-points’, *Addictive Behaviors*, vol. 125.

Wang, X, Qiao, Y & Wang, S 2023, ‘Parental phubbing, problematic smartphone use, and adolescents' learning burnout: A cross-lagged panel analysis’, *Journal of Affective Disorders*, vol. 320, pp. 442-9.

Wu, J & Liu, Q 2023, ‘A longitudinal study on college students' depressive symptoms during the COVID-19 pandemic: The trajectories, antecedents, and outcomes’, *Psychiatry Research*, vol. 321, p. 115058.

Xie, J-Q, Zimmerman, MA, Rost, DH, Yin, X-Q & Wang, J-L 2020, ‘Stressful life events and problematic smartphone usage among Chinese boarding-school adolescents: A moderated mediation model of peer support and depressive symptoms’, *Addiction Research & Theory*, vol. 28, no. 6, pp. 493-500.

Yang, X, Hu, H, Zhao, C, Xu, H, Tu, X & Zhang, G 2021, ‘A longitudinal study of changes in smart phone addiction and depressive symptoms and potential risk factors among Chinese college students’, *BMC Psychiatry*, vol. 21.

Yang, X, Liu, R-D, Ding, Y, Hong, W & Ding, Z 2022, ‘Interpersonal relationships moderate the relation between academic stress and mobile phone addiction via depression among Chinese adolescents: A three-wave longitudinal study’, *Current Psychology*.

Yang, Y, Liu, R-D, Liu, J, Ding, Y, Hong, W & Jiang, S 2022, ‘The Relations between Parental Active Mediation, Parent-Child Relationships and Children's Problematic Mobile Phone Use: a Longitudinal Study’, *Media Psychology*, vol. 25, no. 4, pp. 513-30.

Yoo, SY, Park, SM, Choi, C-H, Chung, SJ, Bhang, S-Y, Kim, J-W, Kweon, Y-S & Choi, J-S 2022, ‘Harm avoidance, daily stress, and problematic smartphone use in children and adolescents’, *Frontiers in Psychiatry*, vol. 13, p. 962189.

Yuan, G, Elhai, JD & Hall, BJ 2021, ‘The influence of depressive symptoms and fear of missing out on severity of problematic smartphone use and internet gaming disorder among Chinese young adults: A three-wave mediation model’, *Addictive Behaviors*, vol. 112.

Yun, J, Han, G & Son, H 2022, ‘Protective and risk factors of problematic smartphone use in preteens using panel study on Korean children’, *Frontiers in Psychiatry*, vol. 13.

Zhang, G, Yang, X, Tu, X, Ding, N & Lau, JTF 2020, ‘Prospective relationships between mobile phone dependence and mental health status among Chinese undergraduate students with college adjustment as a mediator’, *Journal of Affective Disorders*, vol. 260, pp. 498-505.

Zhang, K, Guo, H, Wang, T, Zhang, J, Yuan, G, Ren, J, Zhang, X, Yang, H, Lu, X, Zhu, Z, Du, J, Shi, H, Jin, G, Hao, J, Sun, Y, Su, P & Zhang, Z 2023, ‘A bidirectional association between smartphone addiction and depression among college students: A cross-lagged panel model’, *Frontiers in public health*, vol. 11, p. 1083856.

Zhang, Y, Li, S & Yu, G 2022, ‘The longitudinal relationship between boredom proneness and mobile phone addiction: Evidence from a cross-lagged model’, *Current Psychology*, vol. 41, no. 12, pp. 8821-8.

Zhao, C, Ding, N, Yang, X, Xu, H, Lai, X, Tu, X, Lv, Y, Xu, D & Zhang, G 2021, ‘Longitudinal Effects of Stressful Life Events on Problematic Smartphone Use and the Mediating Roles of Mental Health Problems in Chinese Undergraduate Students’, *Frontiers in public health*, vol. 9.

Zhou, H, Dang, L, Lam, LW, Zhang, MX & Wu, AMS 2021, ‘A cross-lagged panel model for testing the bidirectional relationship between depression and smartphone addiction and the influences of maladaptive metacognition on them in Chinese adolescents’, *Addictive Behaviors*, vol. 120.

Zwilling, M 2022, ‘The Impact of Nomophobia, Stress, and Loneliness on Smartphone Addiction among Young Adults during and after the COVID-19 Pandemic: An Israeli Case Analysis’, *Sustainability*, vol. 14, no. 6.
